# An intersectional analysis of the composite index of anthropometric failures in India

**DOI:** 10.1186/s12939-021-01499-y

**Published:** 2021-07-03

**Authors:** Sabu Ulahannan Kochupurackal, Yogish Channa Basappa, Sangeetha Joice Vazhamplackal, Prashanth N Srinivas

**Affiliations:** 1 Health equity cluster, Institute of Public Health, 3009, II-A Main, 17th Cross, KR Road, Siddanna Layout, Banashankari Stage II, Bengaluru, Karnataka 560070 India; 2 Malabar Medical College, Calicut, Kerala India

**Keywords:** CIAF, Inequality, Intersectionality, NFHS, India

## Abstract

**Background:**

Nutritional inequality in India has been estimated typically using stunting, wasting and underweight separately which hide the overall magnitude and severity of undernutrition. We used the Composite Index of Anthropometric Failure (CIAF) that combines all three forms of anthropometric failures to assess the severity of undernutrition and identify the most vulnerable social groups and geographical hotspots.

**Method:**

CIAF was constructed using child anthropometric data from the fourth round of the National Family Health Survey (NFHS-4, 2015–16). We considered 24 intersecting sub-groups based on intersections across four main axes of inequality i.e., caste [Scheduled Tribe (ST), Scheduled Caste (SC) and Other], economic position (poor and non-poor), place of residence (rural and urban) and gender (male and female) (eg. ST-Poor-Rural-Female). Cross-tabulation and logistic regression were done to assess the odds of CIAF among intersecting groups and to identify the most vulnerable sub-groups. Concentration curve was plotted to visualise economic position inequality in child undernutrition across caste categories. Choropleth maps were constructed and descriptive analysis of the district-level prevalence of CIAF was performed to identify the geographic clustering of undernutrition.

**Results:**

Overall 55.32% of children were undernourished by CIAF and 6.62% of children have simultaneous three anthropometric failure. In sub-group analysis, children from ST and SC caste have a higher risk of undernutrition irrespective of other axis of inequality. Compared with CIAF, economic position inequality was amplified for simultaneous-three-failures among all caste categories. Economic position inequalities within caste are more for other caste and SC categories than with ST. Economic position, caste and gender based inequality in all three failures is more consistent in rural areas than with urban areas. Based on the analysis of the high prevalence in the co-occurrence of two or three failures, 111 districts from 12 of 29 states in India were identified across four geographic clusters.

**Conclusions:**

The study shows social and eco-geographical clustering of multi-dimensional anthropometric failures and indicates the need for focused nutritional interventions among SC and ST community in general and ST children from the poor households. Furthermore, governance interventions that target entire regions across districts and states combined with decentralised planning are needed.

**Supplementary Information:**

The online version contains supplementary material available at 10.1186/s12939-021-01499-y.

## Background

India with 46.6 million children stunted and 25.5 million children wasted contributes to the highest global burden of undernutrition [1]. In terms of prevalence, India has the fifth-highest prevalence of underweight (33.4%) and third-highest prevalence of wasting (17.3%) only after South Sudan (22.7%) and Djibouti (21.5%) [2]. While India bears the highest global burden of child undernutrition, the burden of child undernutrition in India is disproportionately distributed across social groups, economic position, religion, geographical area and gender [3, 4]. Inequality in undernutrition perpetuates other forms of inequality and contributes to intergenerational disadvantage, because of its influence on reduced mental and physical capabilities, and increased risk of morbidities and mortality that reduce the life chances of individuals, hence nutritional inequality is an important public health problem. Nutritional inequalities in India are already well characterised across caste [5, 6], economic position [7, 8], gender [9], and place of residence [10] by single axis. The single-axis analysis of nutritional inequalities, while providing important information on gradients along these axes, does not tell us how undernutrition is distributed within intersecting social groups. For instance, caste by economic position or by place of residence characterisation of these inequalities is unavailable. Indeed, a growing body of work on intersectional theoretical perspective and approach in health inequality research recognises multiple interacting axes of health inequalities such as caste, economic position, gender, disability, migration status etc. often perversely accumulating in particular intersectional sub-groups [3, 11–14]. The interaction of the multiple identities such as caste, economic position, gender and place of residence produces a complex and diverse pattern of health outcomes [12]. Several studies have reported that the burden of child undernutrition in India is disproportionately distributed across the multiple axes of social power such as caste, economic position, religion, and gender [3, 7, 9]. Though intersectional analysis of nutritional inequality by caste, economic position, gender and place of residence [3, 9, 11] and spatial inequality by inter and intra-district inequalities among 640 Indian districts are available [15–18] these analyses have been conducted using anthropometric measures of failure (stunting, wasting and underweight) separately and do not provide a comprehensive estimate of nutritional inequality in India.

While stunting indicates chronic undernutrition, wasting indicates acute undernutrition and underweight is the composite measure of both stunting and wasting [19–21]. According to WHO these three indices reflect distinct biological conditions and any single index cannot be a proxy for these distinct biological phenomena [21]. While categories of under-nutrition based on one of the three indices are helpful diagnostic parameter, a population-level comprehensive estimate of anthropometric failure was needed to account for all population-level co-occurrence of undernutrition [20]. The Composite Index of Anthropometric Failure (CIAF) was developed by Svedberg (2000) as an aggregated indicator of stunting, wasting and underweight [22]. The initial model developed by Svedberg had six subgroups of anthropometric failures (A-F) namely; A- no failure, B- only wasted, C- underweight and wasted, D- stunted, wasted and underweight, E- stunted and underweight, F- only stunted. To these six subgroups, Nandy et al. added one more sub-group Y- only underweight [23]. The CIAF includes the subgroups B-Y and excludes group A with no failure. While it provides an overall magnitude of undernutrition in a population more comprehensively, it also measures the severity of undernutrition by identifying children with multiple anthropometric failures that form the priority group for policy-makers [19, 22, 23]. The Meta-analysis of effects of malnutrition in low-and-middle-income countries shows that children with all three failures have the highest risk of mortality (HR: 12.3, 95% CI::7.7, 19.6) among the undernourished children, combination of two failures, wasted and underweighted was reported more hazardous (HR: 4.7; 95% CI: 3.1, 7.1) than being stunted and underweight (HR: 3.4; 95% CI: 2.6, 4.3) [24]. Hence, from policy and program perspective, CIAF index is an effective instrument for identifying the nutritionally most deprived groups given higher risk of mortality in individuals and populations with co-occurring nutritional failures.

While CIAF reveals the intersections of multiple anthropometric failures, an analysis of CIAF across intersectional sub-groups of caste, economic position, gender and place of residence helps us to identify the most vulnerable social sub-groups with severe forms of undernutrition. Further to this, given the heterogeneous nature of Indian districts in terms of dietary pattern, social norms and economic position inequality, identifying the districts with the highest burden of undernutrition will improve equitable policy formulation and interventions. India’s flagship nutritional program, POSHAN Abhiyaan considers the district as an important sub-unit for action, and district level monitoring is a key element in its implementation. Unlike the previous round of National Family Health Survey (NFHS), the most recent NFHS-4 (2015–16) survey collected unites of samples from district level that enables fine-scale characterisation of undernutrition analysis at the district level. Identification of high-priority districts based on CIAF and intersectional analysis across social groups hold the potential for more comprehensive identification of the high-priority districts and regions. This could also guide targeted and context-specific nutritional interventions and to improve precise financial allocations to the high-priority districts.

## Methods

The data from the fourth round of the National Family Health Survey (NFHS 4, 2015–16) was used to assess the severity of under-nutrition among sub-groups. NFHS is a nationwide survey across a representative sample of households and follows the Demographic and Health Surveys (DHS) system in its use of standardised questionnaires, sample designs, and field procedures. There have been four rounds of NFHS; NFHS-1 (1992–93), NFHS-2 (1998–99), NFHS-3 (2005–06) and NFHS-4 (2015–16). NFHS-4 collected information from a nationally representative sample of 601,509 households and interviewed 699,686 women (age 15–49), and 112,122 men (age 15–54) living in all the 29 states and seven union territories (UTs) of India. Anthropometric data were collected for 259,627 children who stayed in the household the night before the interview. Survey data was collected during the year 2015–16. The survey has calculated height/age, weight/age, weight/height standard deviation based on the new WHO growth chart [25]. Children below − 2 height/age, weight/age, and weight/height standard deviation is categorised as stunted, underweight and wasted respectively. CIAF was constructed using child anthropometric data from National Family Health Survey (NFHS 4, 2015–16).

Concentration curve (cc) was plotted to visualise the pattern and magnitude of economic position inequality in child undernutrition within each caste category. The cumulative percentage of CIAF is plotted on the y-axis and cumulative percentage of wealth status ranked by wealth index on the x-axis. If the concentration curve lies above the 45^0^ line (the line of equality) when the indicator is ill-health [26], it means inequality against the poor and if the curve lies below the line of equality it means the inequality against the rich exist. If the curve is a straight line equal to the line of equality, it depicts perfect equality in child undernutrition irrespective of wealth status and associated concentration index. In order to identify geographical clustering of CIAF, choropleth maps were constructed using GeoDa and descriptive analysis of the district-level prevalence of CIAF was performed. In simultaneous three failures and two failures, we categorised the district as high, medium and low prevalence based on the mean and standard deviation of the district-wise prevalence in each category. The district with a high prevalence of all three failures, simultaneous two failures of wasting and underweight, underweight and stunting were categorised as critical districts for urgent policy interventions. Similarly, high prevalence district in all three failures and either one of the two-dimensional failures were categorised as very serious and high prevalence of two of the two-dimensional failures or only all three failures were categorised as serious districts.

In order to understand the impact of intersecting categories on the nutritional status of children by CIAF, the study followed the intersectional categorisation of Indian social groups by Sen et al. (2009) [27]. For some axes that had multiple sub-categories, these were aggregated/dichotomised. For instance, wealth quintiles were recoded into poor and non-poor; poorest and poorer categories were combined into a single category “poor”, and the middle, richer and richest wealth quintiles were grouped into “non-poor”. In the case of caste, SC and ST were coded separately while OBC and other caste were grouped as Other. Wherever responses for caste were missing (3.4%; 9214 cases) or reported as “don’t know”(0.9%; 1832 cases), these were excluded (4.3% of the total sample) yielding a final sample size of 206,276. From these re-coded categories, twenty-four intersecting sub-groups were created and the prevalence of CIAF for each sub-group calculated (see Table 1). The adjusted odds ratio of CIAF among each of these intersectional groups were calculated using logistic regression keeping Other-Non-Poor-Urban-Male as the reference category. We used 95% Confidence Interval (CI) of odds ratio to infer the significance in the difference between each intersecting sub-groups (based on non-overlapping confidence interval) The odds ratio for each intersectional sub-group were plotted using the ggplot2 package of R statistical software [28].

**Table 1:** Distribution of CIAF by intersecting sub-groups of Caste, Economic position, Gender and Place of Residence

Intersecting Sub-groups	N	%	Only wasting	Underweight and Wasted	Stunted underweighted and wasted	Stunted and Underweighted	Only Stunted	Only underweight	CIAF
ST Poor Female Rural	16,492.00	6.66	6.60	10.80	9.60	24.00	12.70	3.20	66.90
ST Poor Female Urban	727.00	0.29	6.98	7.97	9.07	21.32	15.07	1.56	61.97
ST Poor Male Rural	17,084.00	6.88	5.75	11.76	12.60	23.32	12.71	3.03	69.17
ST Poor Male Urban	770.00	0.31	8.30	9.50	9.00	21.80	16.40	6.90	71.90
ST Non-Poor Female Rural	5503.00	2.21	6.80	7.50	6.80	17.00	12.20	3.00	53.30
ST Non-Poor Female Urban	2881.00	1.16	8.45	8.28	5.76	13.96	11.05	2.18	49.68
ST Non-Poor Male Rural	5762.00	2.32	7.50	8.20	9.10	16.10	13.70	1.90	56.50
ST Non-Poor Male Urban	2980.00	1.20	5.90	12.60	6.40	13.50	11.40	2.50	52.30
SC Poor Female Rural	12,461.00	5.02	4.80	8.30	8.20	26.70	15.40	2.70	66.10
SC Poor Female Urban	1139.00	0.45	7.90	9.40	9.50	23.70	12.10	4.00	66.60
SC Poor Male Rural	13,279.00	5.35	4.50	8.70	10.20	25.20	15.70	2.30	66.60
SC Poor Male Urban	1134.00	0.45	6.59	7.11	8.75	22.38	15.23	7.17	67.23
SC Non-Poor Female Rural	6294.00	2.53	7.46	7.89	3.74	15.01	13.23	2.55	49.88
SC Non-Poor Female Urban	3806.00	1.53	7.10	6.90	4.30	15.70	13.70	2.50	50.20
SC Non-Poor Male Rural	6739.00	2.71	6.10	7.90	6.80	14.40	14.40	2.20	51.80
SC Non-Poor Male Urban	4199.00	1.69	6.34	7.73	5.78	15.38	13.80	2.18	51.21
Other Poor Female Rural	27,055.00	10.90	5.40	8.30	7.20	25.30	15.30	2.80	64.30
Other Poor Female Urban	2501.00	1.00	5.76	7.66	8.24	25.03	12.54	2.12	61.35
Other Poor Male Rural	29,018.00	11.72	5.20	8.30	9.20	23.80	15.10	2.20	63.80
Other Poor Male Urban	2707.00	1.10	5.00	8.80	10.30	23.00	15.10	2.40	64.60
Other Non-Poor Female Rural	23,121.00	9.31	6.70	7.10	4.00	13.40	13.00	2.50	46.70
Other Non-Poor Female Urban	16,921.00	6.80	7.41	7.18	3.72	11.58	11.26	2.55	43.70
Other Non-Poor Male Rural	26,476.00	10.71	6.60	7.80	5.10	13.10	13.10	2.00	47.70
Other Non-Poor Male Urban	19,006.00	7.70	7.10	8.30	4.75	11.49	11.70	2.19	45.53
Total	248,055.00	100.00	6.19	8.22	6.62	18.33	13.41	2.55	55.32

## Results

### Types of anthropometric failures among children

An overall CIAF prevalence of 55.32% among children was found as opposed to 38.4% (stunting), 35.7% (underweight) and 21% (wasting) in the NFHS-4 report. Among the CIAF categories, 22.15% of children suffer from only one form of anthropometric failure (groups B, F and Y), whereas 26.55% of children have simultaneous two failures (groups C and E) and 6.62% have all three forms of anthropometric failures. Simultaneous two failures of stunting and underweight (18.33%; group E) is the highest reported type of anthropometric failure. Overall, children of ST Poor appear disadvantaged both in rural and urban areas compared to SC Poor and Other Poor except for ST Poor Female Urban children; ST Poor Male Rural children have the highest proportion of simultaneous three forms of anthropometric failures (12.60%), whereas the ST Poor Male urban children have the highest proportion of children undernourished (71.90%). Both cross-tabulation of CIAF with wealth quintiles and the intersectional analysis shows that economic position is an important factor affecting the nutritional status of children measured by CIAF. There is a large difference in CIAF between children from the poor and non-poor economic position with non-poor advantage irrespective of gender, caste, and place of residences.

### Inequality in CIAF and all-three failures by economic position within caste groups

**Fig. 1:**
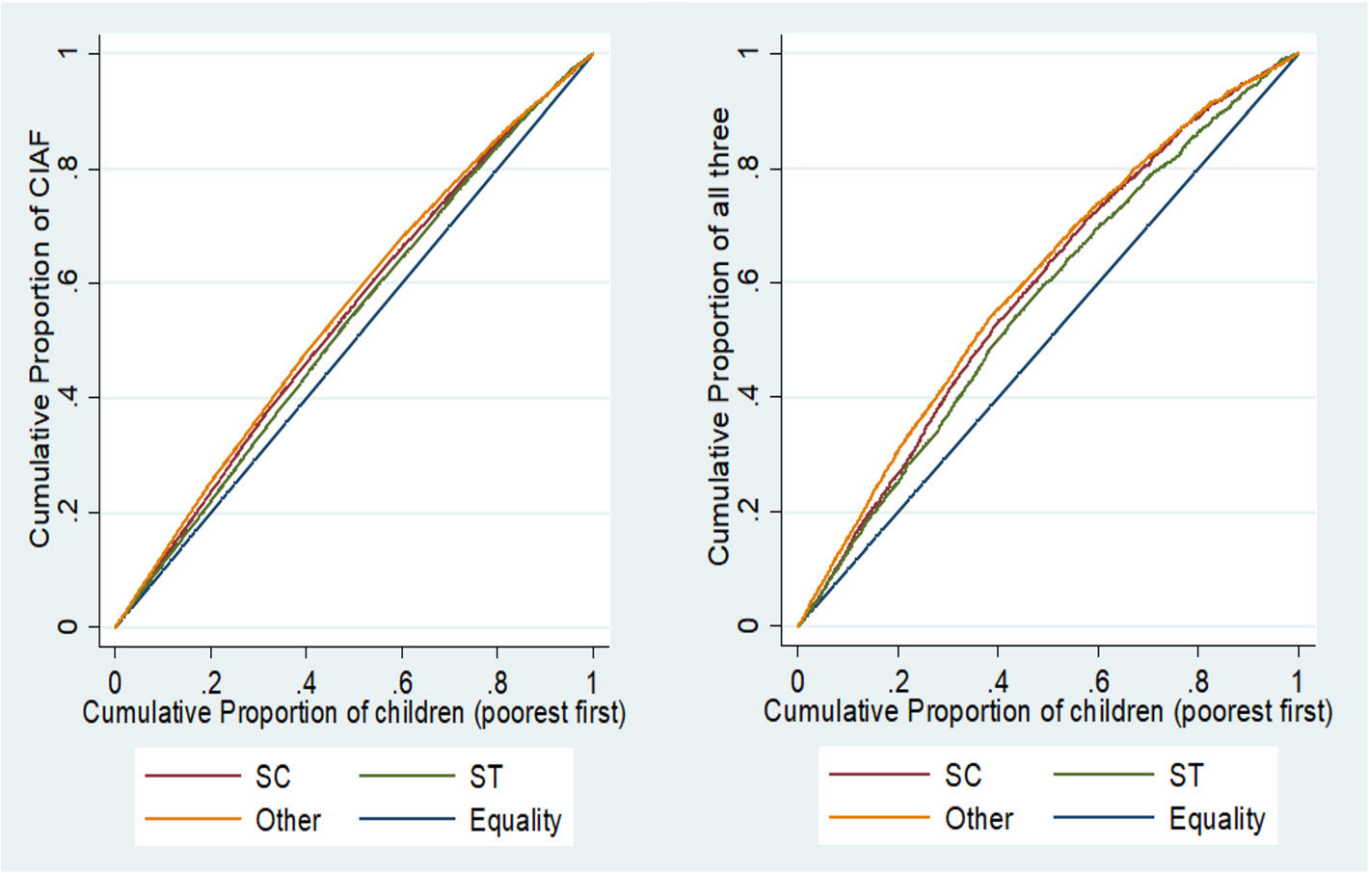
Concentration Curve for CIAF and all three anthropometric failure by Caste Category

Concentration curve (CC) (Fig. 1) shows consistently higher economic position inequality in the severe form of undernutrition (simultaneous three failures) than aggregate CIAF index across all caste groups, indicating relatively more unfair clustering of simultaneous three failures across caste groups than with CIAF. While the highest proportion of children from ST suffered simultaneous three failures, the same community reported the lowest rate of inequality in nutritional status by wealth quintiles. The lack of economic position improvements among the ST community limits our ability to assess the role of economic position in improving the child nutritional status among ST. In OBC and general category (other) where the spread of households across all economic positions is seen, the rich-poor inequalities are higher (compare with ST and SC). Interestingly, the CC of SC demonstrated a shifting trend; while the poorest wealth quintile, of both SC and ST groups, coincide, higher up on the economic position gradient, the SC group makes a departure from this trend and shows increasing inequality and coincides with OBC and general categories. This means that the nature of inequality in child nutritional status among the SC poorest wealth quintiles is similar to that of the ST community whereas, among the upper wealth quintiles the inequality increases further indicating differential effects of economic position improvements within the SC category.

### Intersectional inequality

A cross-tabulation of CIAF and all three failures revealed a significant difference by economic position, caste, gender and place of residence (see additional file 2; Table A). However, when we examined these differences by intersectional sub-groups of caste, economic position, place of residence, and gender, we found that these significant differences were not uniformly distributed. The intersectional sub-group comparison shows that economic position difference in all three failures among ST children is significant only in rural areas, whereas among the urban areas this difference disappears irrespective of gender. Among the SC children, except for urban male children, in all other groups, economic position-based differences appeared significant. Among the other caste groups (OBC and General combined) economic position-based difference is consistently significant irrespective of gender and place of residence. In CIAF, except for ST Female Urban, difference by economic position is consistently significant across all caste group irrespective of gender and place of residence (see Table 2).

**Table 2:** Economic Position Differences in All Three Failures and CIAF by Intersectional Sub-groups

	All three failures			CIAF		
	95% CI			95% CI		
Intersecting Sub-groups	OR	LCI	UCI	OR	LCI	UCI
ST Poor Female Rural	2.07	1.81	2.38	2.37	2.21	2.54
ST Non-Poor Female Rural	1.43	1.08	1.89	1.34	1.18	1.52
ST Poor Female Urban	1.96	0.98	3.93	1.91	1.40	2.61
ST Non-Poor Female Urban	1.2	0.75	1.93	1.17	0.92	1.50
ST Poor Male Rural	2.85	2.51	3.24	2.68	2.5	2.88
ST Non-Poor Male Rural	1.95	1.52	2.52	1.52	1.35	1.72
ST Poor Male Urban	1.94	1.2	3.15	3	2.18	4.12
ST Non-Poor Male Urban	1.33	0.9	1.98	1.29	1.02	1.62
SC Poor Female Rural	1.75	1.53	2	2.29	2.14	2.46
SC Non-Poor Female Rural	0.77	0.63	0.93	1.18	1.09	1.29
SC Poor Female Urban	2.06	1.5	2.83	2.34	1.93	2.84
SC Non-poor Female Urban	0.87	0.65	1.16	1.18	1.04	1.35
SC Poor Male Rural	2.24	1.97	2.54	2.34	2.19	2.51
SC Non-Poor Male Rural	1.42	1.2	1.68	1.26	1.16	1.37
SC Poor Male Urban	1.93	1.44	2.59	2.48	2.03	3.03
SC Non-Poor Male Urban	1.23	0.99	1.52	1.25	1.1	1.42
Other Poor Female Rural	1.53	1.35	1.72	2.12	2	2.24
Other Non-Poor Female Rural	0.82	0.71	0.94	1.03	0.97	1.09
Other Poor Female Urban	1.77	1.34	2.35	1.92	1.63	2.25
Other Non-Poor Female Urban	0.78	0.66	0.92	0.92	0.86	0.99
Other Poor Male Rural	2	1.78	2.24	2.07	1.95	2.19
Other Non-Poor Male Rural	1.05	0.92	1.19	1.07	1.01	1.13
Other Poor Male Urban	2.26	1.79	2.85	2.14	1.85	2.47
Other Non-Poor Male Urban	1			1		

Non-overlapping confidence interval

Marginally overlapping confidence interval

In the caste-based comparison of all three failures, a significant difference between ST and SC in all three failures and CIAF was observed only among poor male rural. At the same time, this difference between ST and other caste is consistently significant only in rural areas irrespective of gender difference. Except for non-poor male rural, in all other sub-groups, the difference in all three failures between SC and other caste is not significant in all three failures. Whereas in CIAF this difference is significant in poor male rural, non-poor female rural, non-poor female urban, non-poor male rural (see additional file 2; Table B). Significant gender difference in all three failure is found only among rural, and in CIAF this significant differences disappeared in all the groups (see additional file 2; Table C). The significant rural and urban difference in all three failures and CIAF disappeared from all its intersectional sub-groups (see additional file 2; Table D).

### District based undernutrition hotspots in India

**Fig. 2:**
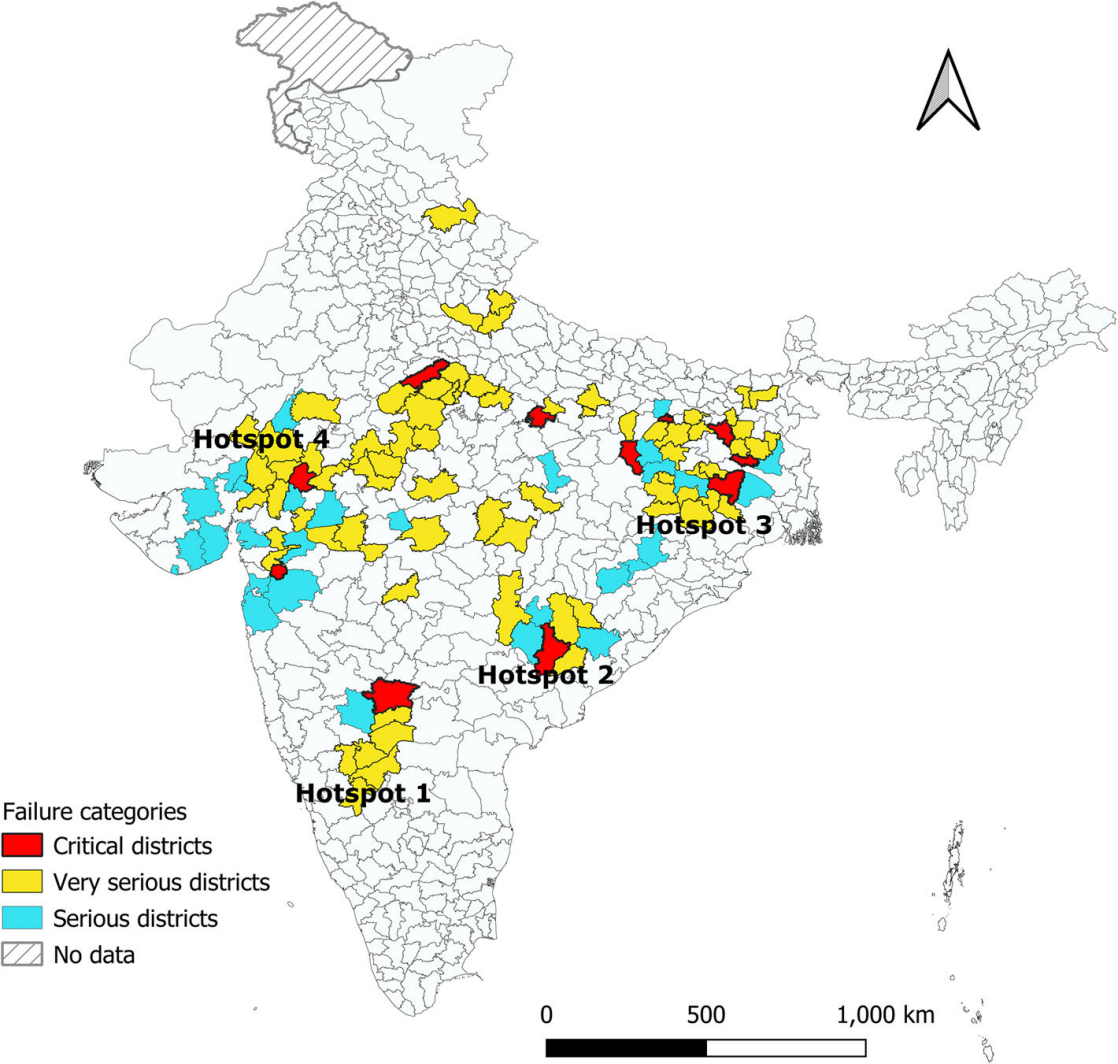
Undernutrition hotspots in India. Map of India showing the undernutrition hotspots; Critical districts = High prevalence in all three failures, stunting and underweight, and underweight and wasting. Very serious districts = High prevalence in all three failures, stunting and underweight or underweight and wasting. Serious districts = High prevalence in all three failures or high prevalence in stunting and underweight, and underweight and wasting

Based on > 1 SD of the mean district prevalence of co-occurring two failures or simultaneous three-failures, (> 23% for stunting and underweight, > 11.7% wasting and underweight, > 9.5% all-three-failures; see additional file 1; Table A), critical (11), very serious (72) and seriously affected (28) districts were identified (see additional file 1; Tables B – G). In all the critical districts, nearly half (> 45%) of children reported at least two simultaneous anthropometric failures. Among these, the Dangs district from Gujarat reported the highest proportion (60.1%) of children with at least two failures. From the states of Bihar and Jharkhand two districts each and from Chhattisgarh, Karnataka, Madhya Pradesh, Rajasthan, Uttar Pradesh, and West Bengal one district each was reported as critical (see additional file 1; Table B and C). Among the very serious districts, Pashchimi Singhbhum district in Jharkhand with 64.7% of children with at least two anthropometric failures nearly met the criteria for being a critical district (underweight and wasting prevalence 11.5%; all-three-failures 21.2%; and stunting and underweight 32%). The highest number of very serious districts are reported from Madhya Pradesh [19], followed by Jharkhand [13] (see additional file 1; Table D and E). Gujarat has the highest number of serious districts [8] followed by Odisha [4] (see additional file 1; Table F and G). Overall spatial distribution of critical, very serious and serious district-level prevalence shows geographical clustering of these districts in four undernutrition hotspots spanning over 12 high burden states. In south India, a cluster of eight districts in north Karnataka forms the undernutrition hotspot. The second undernutrition hotspot is eleven districts along the state boundaries of Chhattisgarh [4], Odisha [6] and Maharashtra [1]. The third hotspot is spread across the regions spanning the borders between West Bengal, Bihar and Jharkhand consisting of 28 districts, of which 11 are from Bihar, 3 from West Bengal and 14 from Jharkhand. The fourth hotspot is 53 districts spanning across Madhya Pradesh [23], Rajasthan [7], Gujarat [17], Maharashtra [4] and Uttar Pradesh [2] (See Fig. 2).

**Fig. 3:**
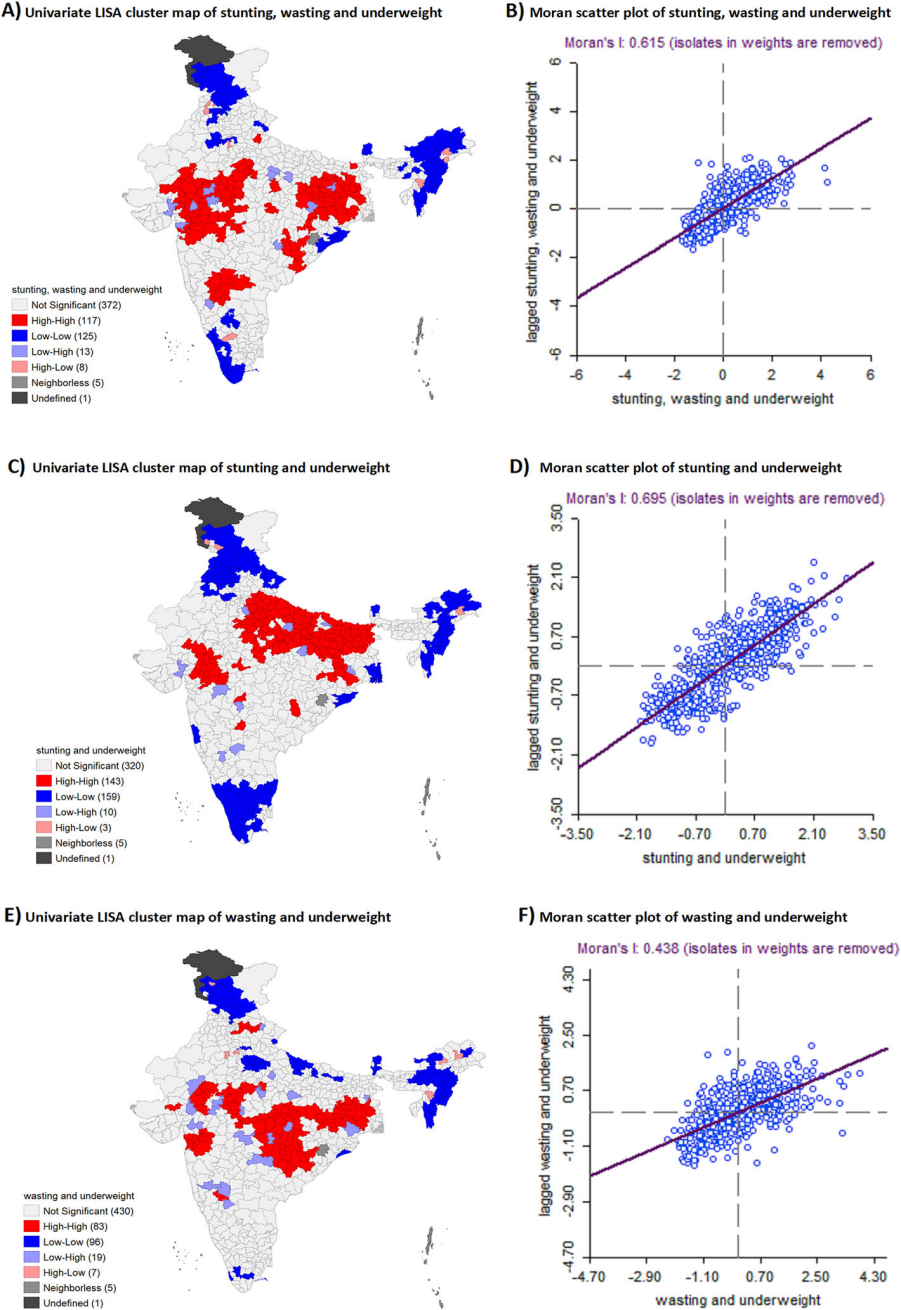
Univariate LISA maps of India showing clustering of undernutrition hotspot and cold spot by two dimensional and three-dimensional anthropometric failures

The Moran plot shows a linear fit through the point cloud. The slope of this line corresponds to Local Moran’s I values were 0.615 for stunting, wasting and underweight, 0.69 for stunning and Underweight, and 0.63 for wasting and underweight (see Fig. 3). All the coefficients were statistically significant (see additional file 2; figure A). This indicates that the three-dimensional and two-dimensional anthropometric failures among children in India is not uniformly distributed across Indian districts, rather there is significant clustering of the high prevalence of two-dimensional and three-dimensional failures in India, further strengthening the case for identifying hotspots.

## Discussion

The novelty of this paper is in its use of CIAF with intersectional analysis to identify sub-groups with the highest overall prevalence and severity of undernutrition and simultaneous anthropometric failures. The clustering of undernutrition across CIAF-based hotspots of two dimensional and three-dimensional anthropometric failures confirms the need for strategies that target larger geographical regions across districts and states.

Previous studies have identified undernutrition hotspot districts for high priority action based three measures of undernutrition separately (stunting, wasting and underweight) [15–18]. Overall, 225 districts have been identified as hotspots based on the high prevalence of three indicators assessed separately [15]. Our study, by applying comprehensive measures of undernutrition and assessing co-occurring failures, identified 111 districts that appeared to be most in need of action. Within in these 111 districts, we have further categorised critical, very serious and seriously affected districts based on the urgency of action. The districts we identify possibly have higher disadvantages due to the clustering of multiple failures. Interestingly, six districts identified as serious (Mahesana and Amreli in Gujarat, Ratlam, Harda, and Shahdol in Madhya Pradesh, and Bankura West Bengal) were not among the undernutrition hotspots identified in previous studies. All the critical and very seriously affected districts in our study have been identified in the single-axis analysis [15].

The clustering of critical, very serious and seriously affected districts also indicates the eco-geographical dimension to the phenomenon of undernutrition. The undernutrition hotspots identified in this study are mostly clustered in the climatic zones of hot and dry (with 40–45 °C during summer days) and composite climatic zones (with 32 – 43 °C during summer days) [29]. The hot and semi-arid climate is also associated with crops cultivation (millets) and other social factors thus requiring interdisciplinary approaches to identify drivers of such clustering [30]. Although hotspot identification is helpful for national-level priority setting, there are several districts in better-off states which continue to have vulnerable groups and undernutrition pockets within districts. For example, in the well-performing state of Kerala, the nutritional status among ST communities is better than the national average of ST communities based on NFHS 4 (2015–16) survey. However, there are the tribal population with a very high prevalence of undernutrition that reported 82.9, 83.6, and 82% of stunting, underweight and wasting among respectively that requires urgent policy attention [31].

The lowest economic position inequality in child nutritional status among the ST group as shown in the CC indicates that the lack of economic position improvements among ST community limits our understanding about the role of economic position in improving the child nutritional status among ST community. As ST population in India is mostly concentrated in rural and remote forested areas [32], significantly higher risk of ST children from rural areas by economic position, caste and gender in undernutrition point towards urgent and more focused intervention among ST children from rural households. The known risk factors of child undernutrition including household poverty [33] lack of access to an improved source of drinking water and toilet facilities, illiteracy, and poor access to health care facilities are higher among ST community compared with other caste groups [34–37]. The impoverished status of ST communities in these determinants suggests that several developmental initiatives in the country to address these were underperforming for tribal groups and requires more community-specific programs.

Shifting CC in all three failures for SC community seems to suggest better social mobility in the SC community compared with the ST community. Consistent with these, previous studies reported that although both SC and ST communities are disadvantaged, SC data indicates higher mobility in income, education, employment status, and political representation [36, 38]. The intersectional analysis shows that nutritional inequality between ST and SC is clustered in rural areas. While economic position based nutritional inequality is more consistently significant in both urban and rural areas for SC and other caste, caste-based nutritional inequality is clustered in rural areas. This could mean that in urban context, households could overcome caste-based disadvantages, whereas economic impediments persist in achieving child nourishment in both rural and urban areas. Rapid urbanisation, combined with rural to urban migrations, mainly of the rural poor, has been associated with increasing inequalities in Indian cities [39]. Increased clustering of urban poor in slums characterised by limited access to public health and nutrition services and amenities [40, 41] along with other known risk factors for undernutrition such as a high density of population, poor quality drinking water, inadequate sanitation facilities, and unhygienic conditions could be the plausible explanation for diminishing the apparent urban advantage for the poor among SC and other castes.

Further, urban poverty too is not uniformly distributed across states; there is a high concentration of urban poor in the poorer states in central and north India, possibly contributing to the greater hotspots in these states [42]. Inter-state and regional variations in urban migration and rural-urban inequalities in child undernutrition in India may reveal different regional patterns.

## Conclusion

Overall, the study shows social and eco-geographical clustering of multi-dimensional anthropometric failures and indicates the need for focused nutritional intervention for SC and ST community in general and ST children from the poor economic position and decentralised planning in nutritional intervention considering socio-demographic and ecological factors. The intersectional analysis of CIAF is useful in bringing out the multiple dimensions of child nutritional inequality. On the one hand, while the CIAF provides biological gradients of severity in undernutrition, the intersectional analysis brings out the multiple dimensions of social identities that underlie the severity of undernutrition. The groups that are disadvantaged on several dimensions, such as poor from SC and ST communities in rural residence need more priority attention. Clustering of critical, very serious and serious districts into undernutrition hotspots suggest the prioritisation of malnutrition hotspots for the nutritional interventions and more decentralised planning considering the eco-geographical factors. The finding also cautions against the policy formulation and programme implementation based on the single axis of inequality. Further investigation of intra-district and state variation in socio-economic inequality in child nutritional status among the caste groups is warranted to design district-specific interventions that can improve equity in child nutritional outcomes.

## Supplementary Information

**Additional file 1.**

**Additional file 2.**

## Data Availability

The data can be downloaded from the website of the Demographic and Health Survey (DHS) (https://dhsprogram.com/data/). The data for the current study were downloaded from the afore-mentioned website.

## Abbreviations

CIAF: Composite Index of Anthropometric Failure
NFHS: National Family Health Survey
ST: Scheduled Tribe
SC: Scheduled Caste
OBC: Other Backward Caste
DHS: Demographic and Health Surveys
WHO: World Health Organisation
CC: Concentration Curve

## References

[1] Fanzo J, Hawkes C, Udomkesmalee E, Afshin A, Allemandi L, Assery O (2018). 2018 Global Nutrition Report: Shining a light to spur action on nutrition.

[2] Unicef (2018). Levels and trends in child malnutrition. eSocialSciences.

[3] Mukhopadhyay S (2016). The apparent non-significance of sex in child undernutrition in India. J Biosoc Sci.

[4] Thorat S, Sabharwal NS. Addressing the unequal burden of malnutrition - Eldis. India Health Beat. 2011;5(5) [cited 2016 Jul 31] Available from: http://www.eldis.org/go/home&id=71591&type=Document#.V52lhqJ7vrk.

[5] Chatterjee K, Sinha RK, Kundu AK, Shankar D, Gope R, Nair N (2016). Social determinants of inequities in under-nutrition (weight-for-age) among under-5 children: a cross sectional study in Gumla district of Jharkhand, India. Int J Equity Health.

[6] Mohan P, Agarwal K, Jain P (2015). Child malnutrition in Rajasthan: study oof tribal migrant Comunities. Econ Polit Wkly.

[7] Chalasani S, Rutstein S (2014). Household wealth and child health in India. Popul Stud.

[8] Subramanian SV, Ackerson LK, Smith GD. Parental BMI and Childhood Undernutrition in India: An Assessment of Intrauterine Influence. Pediatrics. 2010;126(3):e66371. 10.1542/peds.2010-0222. 10.1542/peds.2010-0222 20713473

[9] Joe W, Mishra US, Navaneetham K (2013). Inter-group inequalities in child undernutrition in India: group analogue of the Gini coefficient and Atkinson’s index. Oxf Dev Stud.

[10] Prusty RK, Gouda J, Das S (2014). Nutritional status of preschool children in selected Indian cities: a study of slum and non-slum differentials. Inst Dev Manag.

[11] Mukhopadhyay S (2015). The intersection of gender, caste and class inequalities in child nutrition in rural India. Asian Popul Stud.

[12] Sen G, Iyer A (2016). The mechanisms of intersectioning social inequalities in health. BMJ Glob Health.

[13] Sen G, Iyer A (2012). Who gains, who loses and how: leveraging gender and class intersections to secure health entitlements. Soc Sci Med.

[14] Sen J, Mondal N (2012). Socio-economic and demographic factors affecting the composite index of anthropometric failure (CIAF). Ann Hum Biol.

[15] Khan J, Mohanty SK (2018). Spatial heterogeneity and correlates of child malnutrition in districts of India. BMC Public Health.

[16] Liou L, Kim R, Subramanian SV (2020). Identifying geospatial patterns in wealth disparity in child malnutrition across 640 districts in India. SSM - Popul Health.

[17] Menon P, Headey D, Avula R, Nguyen PH (2018). Understanding the geographical burden of stunting in India: a regression-decomposition analysis of district-level data from 2015–16. Matern Child Nutr.

[18] Singh S, Srivastava S, Upadhyay AK (2019). Socio-economic inequality in malnutrition among children in India: an analysis of 640 districts from National Family Health Survey (2015–16). Int J Equity Health.

[19] Nandy S, Miranda JJ (2008). Overlooking undernutrition? Using a composite index of anthropometric failure to assess how underweight misses and misleads the assessment of undernutrition in young children. Soc Sci Med.

[20] Nandy S, Svedberg P, Preedy VR (2012). The Composite Index of Anthropometric Failure (CIAF): An Alternative Indicator for Malnutrition in Young Children. Handbook of Anthropometry.

[21] WHO (1995). The use and interpretation of anthropometry: report of a WHO expert committee. World Health Organ Tech Rep Ser.

[22] Svedberg P. Poverty and undernutrition: theory, measurement, and policy: Clarendon Press; 2000. 10.1093/0198292686.001.0001.

[23] Nandy S, Irving M, Gordon D, Subramanian SV, Smith GD (2005). Poverty, child undernutrition and morbidity: new evidence from India. Bull World Health Organ.

[24] McDonald CM, Olofin I, Flaxman S, Fawzi WW, Spiegelman D, Caulfield LE (2013). The effect of multiple anthropometric deficits on child mortality: meta-analysis of individual data in 10 prospective studies from developing countries. Am J Clin Nutr.

[25] de Onis M, Garza C, Victora CG, Onyango AW, Frongillo EA, Martines J (2004). The WHO multicentre growth reference study: planning, study design, and methodology. Food Nutr Bull.

[26] Barros AJ, Victora CG (2013). Measuring coverage in MNCH: determining and interpreting inequalities in coverage of maternal, newborn, and child health interventions. PLoS Med.

[27] Sen G, Iyer A, Mukherjee C. A Methodology to Analyse the Intersections of Social Inequalities in Health. J Hum Dev Capab. 2009;10(3):397–415. 10.1080/19452820903048894.

[28] Core Team R (2013). R: a language and environment for statistical computing.

[29] Karakoti I, Das PK (2014). Intercomparability of isotropic and anisotropic solar radiation models for different climatic zones of India. Environ Prog Sustain Energy.

[30] Sanjeev RK, Nuggehalli Srinivas P, Krishnan B, Basappa YC, Dinesh AS, Ulahannan SK (2020). Does cereal, protein and micronutrient availability hold the key to the malnutrition conundrum? An exploratory analysis of cereal cultivation and wasting patterns of India. Wellcome Open Res.

[31] Gangadharan K (2011). Nutritional deprivation of children in rural Kerala an inter caste analysis. IPEDR..

[32] Census. CENSUS OF INDIA 2011. Government of India; 2011 [cited 2016 Mar 17]. Available from: http://censusindia.gov.in/2011-prov-results/data_files/india/paper_contentsetc.pdf

[33] Gang IN, Sen K, Yun M-S (2008). Poverty in rural India: caste and tribe. Rev Income Wealth.

[34] Iversen V, Krishna A, Sen K (2016). Rags to riches? Intergenerational occupational mobility in India.

[35] Mishra US, Joe W (2020). Household assets and wealth quintiles, India 2006–16. Econ Polit Wkly.

[36] Sethi R, Somanathan R. Caste hierarchies and social mobility in India [Internet]. Columbia University; 2010. Available from: http://www.eco.uc3m.es/temp/mobility_may_2010.pdf.

[37] Bang A, Jhalani M, Angami N, Beck H, Jain Y, Kujur JM (2018). Tribal Health in India: Bridging the gap and a roadmap for the future.

[38] Ranganathan T, Tripathi A, Pandey G (2017). Income mobility among social groups. Econ Polit Wkly.

[39] World Bank (2015). World development Indicator (WDI), [internet].

[40] Gupta M, Halim A (2019). The Hidden Epidemic Of Tribal Malnutrition Among Migrants In Rajasthan And Madhya Pradesh.

[41] Yadav P, Dubey BN (2017). Nutritional problems among children in urban slum area. Man India.

[42] Yenneti K, Wei YD, Chen W (2017). The urbanization of poverty in India: Spatio-temporal disparities in consumption expenditures. Geogr Rev.

