# Supplementary material for: An intersectional analysis of the composite index of anthropometric failures in India
Source: Int J Equity Health. 2021 Jul 3;20:155. doi: 10.1186/s12939-021-01499-y (PMC8254924; doi:10.1186/s12939-021-01499-y)
Supplement: Supplementary file 1 — Additional file 1. [file 12939_2021_1499_MOESM1_ESM.pdf]

Table A: Descriptive Statistics of CIAF Categories for 640 Indian Districts

	CIAF	Only Stunting	Only Wasting	Only Underweight	Stunting and underweight	Wasting and underweight	Stunting, underweight and wasting
Mean	52.9	13. 7	6.5	2.3	16.3	8.1	6
Standard Deviation	11.6	4.3	2.9	1.2	6.7	3.6	3.5
Minimum	20.7	5.2	0	0	3	0	0
Maximum	78.6	26.8	19.1	6.7	35.7	22.1	21.2
Low Prevalence	< 41.3	< 9.4	< 3.6	< 1.1	< 9.6	< 4.5	< 2.5
Medium Prevalence	41. 3 - 64.5	9.4 - 18	3.6 - 9.4	1.1 - 3.5	9.6 – 23	4.5 - 11.7	2.5 - 9.5
High Prevalence	64. 5 >	18 >	9.4 >	3.5 >	23 >	11.7 >	9.5 >

Table B: CIAF Prevalence in Critical District

State	District	Only Stunting	Only Wasting	Only Underweight	Stunting and Underweight	Wasting and Underweight	All three	Total
Bihar	Jamui	12.6	7.9	2.3	23.7	11.8	9.9	68.2
Bihar	Arwal	11.3	6.1	2.5	26.5	12.4	12.3	71.0
Chhattisgarh	Dakshin Bastar Dantewada	11.1	6.7	3.1	23.3	15.1	10	69.3
Gujarat	The dangs	8.1	8.2	0.9	24.4	20.6	15.1	77.4
Jharkhand	Garhwa	10.5	5.9	2.8	23.3	12.9	12.4	67.9
Jharkhand	Jamtara	11.7	7.6	3.7	22.9	12.7	9.6	68.2
Karnataka	Gulbarga	12.0	8.8	2.8	28.4	13.2	12	77.2
Madhya Pradesh	Morena	10.0	7.2	2.4	27.2	12.2	10.4	69.4
Rajasthan	Banswara	13.3	6.5	2.1	24.8	12.2	12.3	71.2
Uttar Pradesh	Chitrakoot	12.8	6.9	2.7	23	11.8	14.9	72.0
West Bengal	Puruliya	9.1	4.4	4.6	23	17.7	12.6	71.4

Table C: CIAF Prevalence category in Critical District								
		Only Stunting	Only Wasting	Only Underweight	Stunting and Underweight	Wasting and Underweight	All three	CIAF
Bihar	Jamui	Medium	Medium	Medium	High	High	High	High
Bihar	Arwal	Medium	Medium	Medium	High	High	High	High
Chhattisgarh	Dakshin Bastar Dantewada	Medium	Medium	Medium	High	High	High	High
Gujarat	The dangs	Low	Medium	Low	High	High	High	High
Jharkhand	Garhwa	Medium	Medium	Medium	High	High	High	High
Jharkhand	Jamtara	Medium	Medium	High	High	High	High	High
Karnataka	Gulbarga	Medium	Medium	Medium	High	High	High	High
Madhya Pradesh	Morena	Medium	Medium	Medium	High	High	High	High
Rajasthan	Banswara	Medium	Medium	Medium	High	High	High	High
Uttar Pradesh	Chitrakoot	Medium	Medium	Medium	High	High	High	High
West Bengal	Puruliya	Low	Medium	High	High	High	High	High

Table D: CIAF prevalence % in very serious districts

State	District	Only Stunting	Only Wasting	Only Underweight	Stunting and Underweight	Wasting and Underweight	All three	CIAF
Bihar	Purnia	14.3	3.5	2.0	27.7	7.3	10.3	65.1
Bihar	Madhepura	13.8	4.9	2.0	27.6	8.7	10.8	67.7
Bihar	Banka	14.1	5.4	2.5	25.5	10.3	10.3	68.1
Bihar	Munger	13.2	4.6	4.8	23.6	6.4	10	62.6
Bihar	Nalanda	16.3	4.1	2.7	26.6	8.8	11.3	69.9
Bihar	kaimur (bhabua)	14.3	4.5	2.2	29.3	6.4	10.9	67.6
Bihar	Aurangabad	10.3	7.2	2.8	26.7	7.1	10.6	64.7
Bihar	Gaya	11.8	4.3	3.5	28.4	8.8	12.7	69.5
Bihar	Nawada	12.9	4.3	3.3	25.9	6.8	10.2	63.5
Chhattisgarh	Bastar	9.9	6.9	3.8	19.6	14.6	12.3	67.1
Gujarat	Panchmahal	12.5	13.3	1.6	16.7	12	11.6	67.8

Gujarat	Narmada	11.8	6.4	3.7	21	14.1	15.5	72.5
Gujarat	Tapi	10.4	9.6	2.9	13.4	14.6	12.2	63.1
Gujarat	Sabarkantha	13.7	4.7	2.4	24.9	6.1	12.6	64.4
Gujarat	Kheda	10.3	5.3	3.8	23.3	10	12.1	64.8
Jharkhand	Bokaro	7.3	7.9	2.1	20	16.6	12.7	66.7
Jharkhand	Lohardaga	9.0	6.7	3.7	22.2	12.1	10.3	64.0
Jharkhand	purbi singhbhum	8.7	9.0	2.5	15.1	15.9	15.8	67.0
Jharkhand	Ramgarh	9.2	6.7	4.8	18	12.1	11.7	62.5
Jharkhand	Dumka	10.2	9.3	2.4	18.6	17.4	14.4	72.4
Jharkhand	Khunti	8.9	8.8	2.4	17	19.8	14.5	71.3
Jharkhand	Gumla	12.9	6.1	3.1	19.7	11.8	14	67.6
Jharkhand	Simdega	10.1	10.5	3.4	18.5	14.9	11.5	68.8
Jharkhand	Chatra	11.9	7.5	2.7	25.1	9.6	12.7	69.5
Jharkhand	Deoghar	11.6	4.5	3.2	23.6	9	9.8	61.7
Jharkhand	Pakur	14.7	6.7	3.1	27	6.5	10.9	68.9
Jharkhand	Pashchimi Singhbhum	6.6	5.4	2.0	32	11.5	21.2	78.7
Jharkhand	Saraikela Kharsawan	7.7	3.0	4.6	27.7	10.5	9.8	63.3
Karnataka	Raichur	13.1	11.0	3.3	13.9	13.9	10.5	65.7
Karnataka	Gadag	12.0	16.6	0.8	11.8	13.4	13	67.5
Karnataka	Koppal	18.0	3.8	2.5	26.2	7.8	13.9	72.2
Karnataka	Bellary	11.4	6.5	4.8	29.3	9.7	9.6	71.3
Karnataka	Davanagere	13.7	5.6	2.2	23.9	7	10	62.5
Karnataka	Yadgir	13.3	8.3	1.1	25.5	7.1	16	71.3
Madhya Pradesh	Gwalior	10.6	5.3	3.6	22.2	12.1	10.2	64.0
Madhya Pradesh	Rajgarh	9.4	6.3	3.4	17.7	13.8	12.5	63.0
Madhya Pradesh	Betul	5.9	8.5	3.4	15.8	13.3	12.4	59.4
Madhya Pradesh	Dindori	14.1	5.0	2.3	22.1	12.3	9.7	65.5
Madhya Pradesh	Seoni	9.1	7.2	3.8	14.9	15.9	10.4	61.3
Madhya Pradesh	Balaghat	7.3	8.8	2.3	15.7	14.4	9.6	58.0
Madhya Pradesh	Guna	10.2	5.4	2.6	20.5	15.3	12.2	66.2
Madhya Pradesh	Ashoknagar	11.3	6.6	2.9	19.6	11.9	12.1	64.4

Madhya Pradesh	Sheopur	9.8	2.8	2.9	27.7	10.3	14.9	68.4
Madhya Pradesh	Bhind	9.9	6.8	2.4	23.9	9.8	13.8	66.6
Madhya Pradesh	Datia	13.5	6.6	1.8	25.9	9	10	66.8
Madhya Pradesh	Shivpuri	13.9	4.9	4.5	23.7	10.5	10.4	67.9
Madhya Pradesh	Ratlam	12.4	5.0	2.2	23.1	7.1	10	59.8
Madhya Pradesh	Shajapur	13.7	9.0	3.1	24.7	10.5	10	71.0
Madhya Pradesh	Khargone (west nimar)	13.8	4.5	3.5	24.7	6.9	10	63.4
Madhya Pradesh	Barwani	9.8	6.8	3.4	30.5	9.7	11.5	71.6
Madhya Pradesh	Raisen	13.1	5.2	1.9	23.3	9.8	9.5	62.8
Madhya Pradesh	Alirajpur	9.5	9.2	2.6	26.5	10.3	13.3	71.3
Madhya Pradesh	Burhanpur	14.4	3.0	3.2	25.6	7.4	9.6	63.1
Maharashtra	Washim	11.2	9.0	1.6	17.9	12.3	11.2	63.3
Maharashtra	Gadchiroli	11.3	14.2	1.5	8.8	20.2	12.1	68.2
Odisha	Nabarangapur	11.1	6.9	2.8	18.8	13.5	15.1	68.1
Odisha	Malkangiri	11.1	5.0	3.0	21.4	13.7	13.5	67.7
Rajasthan	Sirohi	9.0	10.8	2.8	21.1	14.6	11.7	70.0
Rajasthan	Bhilwara	8.2	11.5	2.2	16.3	12.9	10.1	61.3
Rajasthan	Dungarpur	10.0	7.5	1.8	21.8	14.6	15.7	71.4
Rajasthan	Jhalawar	10.7	5.5	2.9	18	16.6	9.8	63.5
Rajasthan	Pratapgarh	9.7	8.1	3.8	21	14.9	15.3	72.8
Rajasthan	Udaipur	10.5	6.2	3.5	24.9	11.5	12	68.6
Uttar Pradesh	Jalaun	12.1	5.4	0.7	21.7	14.7	11.8	66.4
Uttar Pradesh	Hamirpur	12.2	10.7	1.2	17.1	12.9	10	64.1
Uttar Pradesh	Budaun	11.0	3.3	3.0	33.8	6.8	10.2	68.0
Uttar Pradesh	Pilibhit	16.6	3.7	2.2	23.6	6.8	11.5	64.4
Uttar Pradesh	Shahjahanpur	8.6	2.7	4.2	29.5	9.2	11.7	65.8
Uttar Pradesh	Kaushambi	11.4	8.2	3.9	26.9	10.2	11.8	72.4
Uttar Pradesh	Jaunpur	8.8	4.1	3.4	26.6	10.4	13	66.3
Uttar Pradesh	sant ravidas nagar (bhadohi)	12.6	3.3	2.6	28.5	8	10.7	65.6
Uttarakhand	Uttarkashi	14.1	10.7	0.8	10.3	18.4	10.7	65.0

Table E: CIAF prevalence category in very serious districts								
State	District	Only Stunting	Only Wasting	Only Underweight	Stunting and Underweight	Wasting and Underweight	All three	CIAF
Bihar	Purnia	Medium	Low	Medium	High	Medium	High	High
Bihar	Madhepura	Medium	Medium	Medium	High	Medium	High	High
Bihar	Banka	Medium	Medium	Medium	High	Medium	High	High
Bihar	Munger	Medium	Medium	High	High	Medium	High	Medium
Bihar	Nalanda	Medium	Medium	Medium	High	Medium	High	High
Bihar	kaimur (bhabua)	Medium	Medium	Medium	High	Medium	High	High
Bihar	Aurangabad	Medium	Medium	Medium	High	Medium	High	High
Bihar	Gaya	Medium	Medium	Medium	High	Medium	High	High
Bihar	Nawada	Medium	Medium	Medium	High	Medium	High	Medium
Chhattisgarh	Bastar	Medium	Medium	High	medium	High	High	High
Gujarat	Panchmahal	Medium	High	Medium	medium	High	High	High
Gujarat	Narmada	Medium	Medium	High	medium	High	High	High
Gujarat	Tapi	Medium	High	Medium	medium	High	High	Medium
Gujarat	Sabarkantha	Medium	Medium	Medium	High	Medium	High	Medium
Gujarat	Kheda	Medium	Medium	High	High	Medium	High	High
Jharkhand	Bokaro	Low	Medium	Medium	medium	High	High	High
Jharkhand	Lohardaga	Low	Medium	High	medium	High	High	Medium
Jharkhand	purbi singhbhum	Low	Medium	Medium	medium	High	High	High
Jharkhand	Ramgarh	Low	Medium	High	medium	High	High	Medium
Jharkhand	Dumka	Medium	Medium	Medium	medium	High	High	High
Jharkhand	Khunti	Low	Medium	Medium	medium	High	High	High
Jharkhand	Gumla	Medium	Medium	Medium	medium	High	High	High
Jharkhand	Simdega	Medium	High	Medium	medium	High	High	High
Jharkhand	Chatra	Medium	Medium	Medium	High	Medium	High	High
Jharkhand	Deoghar	Medium	Medium	Medium	High	Medium	High	Medium
Jharkhand	Pakur	Medium	Medium	Medium	High	Medium	High	High
Jharkhand	Pashchimi Singhbhum	Low	Medium	Medium	High	Medium	High	High
Jharkhand	Saraikela Kharsawan	Low	Low	High	High	Medium	High	Medium

Karnataka	Raichur	Medium	High	Medium	medium	High	High	High
Karnataka	Gadag	Medium	High	Low	medium	High	High	High
Karnataka	Koppal	Medium	Medium	Medium	High	Medium	High	High
Karnataka	Bellary	Medium	Medium	High	High	Medium	High	High
Karnataka	Davanagere	Medium	Medium	Medium	High	Medium	High	Medium
Karnataka	Yadgir	Medium	Medium	Low	High	Medium	High	High
Madhya Pradesh	Gwalior	Medium	Medium	High	medium	High	High	Medium
Madhya Pradesh	Rajgarh	Low	Medium	Medium	medium	High	High	Medium
Madhya Pradesh	Betul	Low	Medium	Medium	medium	High	High	Medium
Madhya Pradesh	Dindori	Medium	Medium	Medium	medium	High	High	High
Madhya Pradesh	Seoni	Low	Medium	High	medium	High	High	Medium
Madhya Pradesh	Balaghat	Low	Medium	Medium	medium	High	High	Medium
Madhya Pradesh	Guna	Medium	Medium	Medium	medium	High	High	High
Madhya Pradesh	Ashoknagar	Medium	Medium	Medium	medium	High	High	Medium
Madhya Pradesh	Sheopur	Medium	Low	Medium	High	Medium	High	High
Madhya Pradesh	Bhind	Medium	Medium	Medium	High	Medium	High	High
Madhya Pradesh	Datia	Medium	Medium	Medium	High	Medium	High	High
Madhya Pradesh	Shivpuri	Medium	Medium	High	High	Medium	High	High
Madhya Pradesh	Ratlam	Medium	Medium	Medium	High	Medium	High	Medium
Madhya Pradesh	Shajapur	Medium	Medium	Medium	High	Medium	High	High
Madhya Pradesh	Khargone (west nimar)	Medium	Medium	Medium	High	Medium	High	Medium
Madhya Pradesh	Barwani	Medium	Medium	Medium	High	Medium	High	High
Madhya Pradesh	Raisen	Medium	Medium	Medium	High	Medium	High	Medium
Madhya Pradesh	Alirajpur	Medium	Medium	Medium	High	Medium	High	High
Madhya Pradesh	Burhanpur	Medium	Low	Medium	High	Medium	High	Medium
Maharashtra	Washim	Medium	Medium	Medium	medium	High	High	Medium
Maharashtra	Gadchiroli	Medium	High	Medium	Low	High	High	High
Odisha	Nabarangapur	Medium	Medium	Medium	medium	High	High	High
Odisha	Malkangiri	Medium	Medium	Medium	medium	High	High	High
Rajasthan	Sirohi	Low	High	Medium	medium	High	High	High
Rajasthan	Bhilwara	Low	High	Medium	medium	High	High	Medium

Rajasthan	Dungarpur	Medium	Medium	Medium	medium	High	High	High
Rajasthan	Jhalawar	Medium	Medium	Medium	medium	High	High	Medium
Rajasthan	Pratapgarh	Medium	Medium	High	medium	High	High	High
Rajasthan	Udaipur	Medium	Medium	Medium	High	Medium	High	High
Uttar Pradesh	Jalaun	Medium	Medium	Low	medium	High	High	High
Uttar Pradesh	Hamirpur	Medium	High	Medium	medium	High	High	Medium
Uttar Pradesh	Budaun	Medium	Low	Medium	High	Medium	High	High
Uttar Pradesh	Pilibhit	Medium	Medium	Medium	High	Medium	High	Medium
Uttar Pradesh	Shahjahanpur	Low	Low	High	High	Medium	High	High
Uttar Pradesh	Kaushambi	Medium	Medium	High	High	Medium	High	High
Uttar Pradesh	Jaunpur	Low	Medium	Medium	High	Medium	High	High
Uttar Pradesh	sant ravidas nagar (bhadohi)	Medium	Low	Medium	High	Medium	High	High
Uttarakhand	Uttarkashi	Medium	High	Low	medium	High	High	High

Table F: CIAF prevalence in serious districts

Name of the	Name of	Only Stunting	Only Wasting	Only Underweight	Stunting and Underweight	Wasting and Underweight	All three	CIAF
Bihar	Bhojpur	11.7	5.2	2.6	23.9	12.9	8.2	64.5
Chhattisgarh	Narayanpur	17.5	8.2	1.8	25	16.4	6	74.9
Chhattisgarh	Bijapur	16.4	8.7	3.0	26.8	12.4	4.7	72.0
Gujarat	Mahesana	11.6	4.0	4.0	17	9	12.6	58.2
Gujarat	Gandhinagar	7.5	6.8	3.8	16.5	10.9	11.8	57.3
Gujarat	Amreli	12.3	8.1	1.2	14.9	5	10.1	51.6
Gujarat	Bhavnagar	14.9	6.2	1.9	22.6	8.6	10.8	65.0
Gujarat	Bharuch	8.0	8.7	3.1	20.5	8.3	12.6	61.2
Gujarat	Valsad	12.0	8.4	1.6	18.9	9.3	12.7	62.9

Gujarat	Surendranagar	15.6	6.6	2.5	23.3	12.5	7.9	68.4
Gujarat	Dohad	8.8	6.1	3.6	29	12.7	5.6	65.8
Jharkhand	Palamu	12.2	4.9	4.3	21	6.3	12.4	61.1
Jharkhand	Latehar	13.1	6.1	2.5	19.1	11.4	11.8	63.9
Jharkhand	Ranchi	10.8	7.2	3.4	20	10.3	10.2	61.8
Karnataka	Bijapur	16.5	9.3	1.6	16.4	7.2	11.5	62.5
Madhya Pradesh	Dhar	9.5	9.2	3.5	21.9	10.9	11.1	66.1
Madhya Pradesh	Harda	11.9	5.4	2.8	18	9.8	9.7	57.6
Madhya Pradesh	Shahdol	10.2	7.0	3.4	17.2	11.3	9.7	58.8
Maharashtra	Nandurbar	6.1	8.4	4.7	20.5	10.2	20.7	70.5
Maharashtra	Nashik	11.7	11.7	1.4	20	10	11	65.8
Maharashtra	Thane	11.0	8.7	2.1	18.2	10.7	9.8	60.5
Odisha	Sambalpur	7.6	5.9	2.8	19.9	10.6	12.6	59.4
Odisha	Subarnapur	17.0	4.1	4.3	20	8.8	9.9	64.2
Odisha	Balangir	14.8	4.4	5.3	17.7	9.8	12.3	64.3
Odisha	Koraput	9.1	6.0	3.6	18.2	9.8	13	59.7
Rajasthan	Rajsamand	13.0	8.4	2.6	15.9	10.4	9.7	60.0
West Bengal	Birbhum	10.2	8.3	1.6	20.5	11.3	9.7	61.6
West Bengal	Bankura	9.0	5.6	4.8	13.1	10.3	11.1	54.0

Table G: CIAF prevalence category in serious districts

Name of the	Name of	Only Stunting	Only Wasting	Only Underweight	Stunting and Underweight	Wasting and Underweight	All three	CIAF
Bihar	Bhojpur	Medium	Medium	Medium	High	High	Medium	Medium
Chhattisgarh	Narayanpur	Medium	Medium	Medium	High	High	Medium	High
Chhattisgarh	Bijapur	Medium	Medium	Medium	High	High	Medium	High
Gujarat	Mahesana	Medium	Medium	High	medium	Medium	High	Medium
Gujarat	Gandhinagar	Low	Medium	High	medium	Medium	High	Medium

Gujarat	Amreli	Medium	Medium	Medium	medium	Medium	High	Medium
Gujarat	Bhavnagar	Medium	Medium	Medium	medium	Medium	High	High
Gujarat	Bharuch	Low	Medium	Medium	medium	Medium	High	Medium
Gujarat	Valsad	Medium	Medium	Medium	medium	Medium	High	Medium
Gujarat	Surendranagar	Medium	Medium	Medium	High	High	Medium	High
Gujarat	Dohad	Low	Medium	High	High	High	Medium	High
Jharkhand	Palamu	Medium	Medium	High	medium	Medium	High	Medium
Jharkhand	Latehar	Medium	Medium	Medium	medium	Medium	High	Medium
Jharkhand	Ranchi	Medium	Medium	Medium	medium	Medium	High	Medium
Karnataka	Bijapur	Medium	Medium	Medium	medium	Medium	High	Medium
Madhya Pradesh	Dhar	Medium	Medium	Medium	medium	Medium	High	High
Madhya Pradesh	Harda	Medium	Medium	Medium	medium	Medium	High	Medium
Madhya Pradesh	Shahdol	Medium	Medium	Medium	medium	Medium	High	Medium
Maharashtra	Nandurbar	Low	Medium	High	medium	Medium	High	High
Maharashtra	Nashik	Medium	High	Medium	medium	Medium	High	High
Maharashtra	Thane	Medium	Medium	Medium	medium	Medium	High	Medium
Odisha	Sambalpur	Low	Medium	Medium	medium	Medium	High	Medium
Odisha	Subarnapur	Medium	Medium	High	medium	Medium	High	Medium
Odisha	Balangir	Medium	Medium	High	medium	Medium	High	Medium
Odisha	Koraput	Low	Medium	High	medium	Medium	High	Medium
Rajasthan	Rajsamand	Medium	Medium	Medium	medium	Medium	High	Medium
West Bengal	Birbhum	Medium	Medium	Medium	medium	Medium	High	Medium
West Bengal	Bankura	Low	Medium	High	medium	Medium	High	Medium
