# Supplementary material for: An intersectional analysis of the composite index of anthropometric failures in India
Source: Int J Equity Health. 2021 Jul 3;20:155. doi: 10.1186/s12939-021-01499-y (PMC8254924; doi:10.1186/s12939-021-01499-y)

Table A: Inequality in the prevalence of CIAF and all three failures by economic position, caste, gender, and place of residence

	CIAF %	95% CI LCI	95% CI UCI	std error	Three failure %	95% CI LCI	95% CI UCI	std error
Place of residence								
Urban	48.32	47.92	48.72	0.20	5.09	4.91	5.26	0.09
Rural	58.03	57.78	58.27	0.13	7.21	7.08	7.34	0.07
Sex of child								
Male	55.65	55.36	55.94	0.15	7.42	7.27	7.58	0.08
Female	54.98	54.68	55.28	0.15	5.76	5.62	5.90	0.07
Caste								
SC	59.28	58.84	59.73	0.23	7.38	7.14	7.62	0.12
ST	63.99	63.35	64.61	0.32	9.97	9.58	10.37	0.20
Other	52.93	52.66	53.19	0.13	5.92	5.80	6.05	0.06
Wealth Index								
Poorest	68.48	68.09	68.87	0.20	9.91	9.66	10.17	0.13
Poorer	60.48	60.04	60.92	0.22	7.61	7.38	7.85	0.12
Middle	53.40	52.92	53.87	0.24	5.83	5.61	6.05	0.11
Richer	46.35	45.86	46.84	0.25	4.54	4.34	4.75	0.10
Richest	38.88	38.35	39.42	0.27	3.16	2.98	3.36	0.10

Table B: Caste Differences in All Three Failures in Intersectional Subgroup Comparison

	All three failures			CIAF		
		95% CI			95% CI	
Intersecting Sub-groups	OR	LCI	UCI	OR	LCI	UCI
ST Poor Female Rural	2.07	1.81	2.38	2.37	2.21	2.54
SC Poor Female Rural	1.75	1.53	2.00	2.29	2.14	2.46
ST Poor Female Urban	1.96	0.98	3.93	1.91	1.40	2.61
SC Poor Female Urban	2.06	1.50	2.83	2.34	1.93	2.84
ST Poor Male Rural	2.85	2.51	3.24	2.68	2.50	2.88
SC Poor Male Rural	2.24	1.97	2.54	2.34	2.19	2.51
ST Poor Male Urban	1.94	1.20	3.15	3.00	2.18	4.12
SC Poor Male Urban	1.93	1.44	2.59	2.48	2.03	3.03
ST Non-Poor Female Rural	1.43	1.08	1.89	1.34	1.18	1.52
SC Non-Poor Female Rural	0.77	0.63	0.93	1.18	1.09	1.29
ST Non-Poor Female Urban	1.20	0.75	1.93	1.17	0.92	1.50
SC Non-poor Female Urban	0.87	0.65	1.16	1.18	1.04	1.35
ST Non-Poor Male Rural	1.95	1.52	2.52	1.52	1.35	1.72

SC Non-Poor Male Rural	1.42	1.20	1.68	1.26	1.16	1.37
ST Non-Poor Male Urban	1.33	0.90	1.98	1.29	1.02	1.62
SC Non-Poor Male Urban	1.23	0.99	1.52	1.25	1.10	1.42
ST Poor Female Rural	2.07	1.81	2.38	2.37	2.21	2.54
Other Poor Female Rural	1.53	1.35	1.72	2.12	2.00	2.24
ST Poor Female Urban	1.96	0.98	3.93	1.91	1.40	2.61
Other Poor Female Urban	1.77	1.34	2.35	1.92	1.63	2.25
ST Poor Male Rural	2.85	2.51	3.24	2.68	2.50	2.88
Other Poor Male Rural	2.00	1.78	2.24	2.07	1.95	2.19
ST Poor Male Urban	1.94	1.20	3.15	3.00	2.18	4.12
Other Poor Male Urban	2.26	1.79	2.85	2.14	1.85	2.47
ST Non-Poor Female Rural	1.43	1.08	1.89	1.34	1.18	1.52
Other Non-Poor Female Rural	0.82	0.71	0.94	1.03	0.97	1.09
ST Non-Poor Female Urban	1.20	0.75	1.93	1.17	0.92	1.50
Other Non-Poor Female Urban	0.78	0.66	0.92	0.92	0.86	0.99
ST Non-Poor Male Rural	1.95	1.52	2.52	1.52	1.35	1.72
Other Non-Poor Male Rural	1.05	0.92	1.19	1.07	1.01	1.13
ST Non-Poor Male Urban	1.33	0.90	1.98	1.29	1.02	1.62
Other Non-Poor Male Urban	1.00			1.00		
SC Poor Female Rural	1.75	1.53	2.00	2.29	2.14	2.46
Other Poor Female Rural	1.53	1.35	1.72	2.12	2.00	2.24
SC Poor Female Urban	2.06	1.50	2.83	2.34	1.93	2.84
Other Poor Female Urban	1.77	1.34	2.35	1.92	1.63	2.25
SC Poor Male Rural	2.24	1.97	2.54	2.34	2.19	2.51
Other Poor Male Rural	2.00	1.78	2.24	2.07	1.95	2.19
SC Poor Male Urban	1.93	1.44	2.59	2.48	2.03	3.03
Other Poor Male Urban	2.26	1.79	2.85	2.14	1.85	2.47
SC Non-Poor Female Rural	0.77	0.63	0.93	1.18	1.09	1.29
Other Non-Poor Female Rural	0.82	0.71	0.94	1.03	0.97	1.09
SC Non-poor Female Urban	0.87	0.65	1.16	1.18	1.04	1.35
Other Non-Poor Female Urban	0.78	0.66	0.92	0.92	0.86	0.99
SC Non-Poor Male Rural	1.42	1.20	1.68	1.26	1.16	1.37
Other Non-Poor Male Rural	1.05	0.92	1.19	1.07	1.01	1.13
SC Non-Poor Male Urban	1.23	0.99	1.52	1.25	1.10	1.42
Other Non-Poor Male Urban	1.00			1.00		

Non-overlapping confidence interval

Marginally overlapping confidence interval

Table C: Male v/s Female Intersectional Subgroup Comparison in All three failures and CIAF

	All three failures			CIAF		
		95% CI			95% CI	
Intersecting Sub-groups	OR	LCI	UCI	OR	LCI	UCI
ST Poor Female Rural	2.07	1.81	2.38	2.37	2.21	2.54
ST Poor Male Rural	2.85	2.51	3.24	2.68	2.5	2.88
ST Poor Female Urban	1.96	0.98	3.93	1.91	1.4	2.61
ST Poor Male Urban	1.94	1.2	3.15	3	2.18	4.12
ST Non-Poor Female Rural	1.43	1.08	1.89	1.34	1.18	1.52
ST Non-Poor Male Rural	1.95	1.52	2.52	1.52	1.35	1.72
ST Non-Poor Female Urban	1.2	0.75	1.93	1.17	0.92	1.5
ST Non-Poor Male Urban	1.33	0.9	1.98	1.29	1.02	1.62
SC Poor Female Rural	1.75	1.53	2	2.29	2.14	2.46
SC Poor Male Rural	2.24	1.97	2.54	2.34	2.19	2.51
SC Poor Female Urban	2.06	1.5	2.83	2.34	1.93	2.84
SC Poor Male Urban	1.93	1.44	2.59	2.48	2.03	3.03
SC Non-Poor Female Rural	0.77	0.63	0.93	1.18	1.09	1.29
SC Non-Poor Male Rural	1.42	1.2	1.68	1.26	1.16	1.37
SC Non-poor Female Urban	0.87	0.65	1.16	1.18	1.04	1.35
SC Non-Poor Male Urban	1.23	0.99	1.52	1.25	1.1	1.42
Other Poor Female Rural	1.53	1.35	1.72	2.12	2	2.24
Other Poor Male Rural	2	1.78	2.24	2.07	1.95	2.19
Other Poor Female Urban	1.77	1.34	2.35	1.92	1.63	2.25
Other Poor Male Urban	2.26	1.79	2.85	2.14	1.85	2.47
Other Non-Poor Female Rural	0.82	0.71	0.94	1.03	0.97	1.09
Other Non-Poor Male Rural	1.05	0.92	1.19	1.07	1.01	1.13
Other Non-Poor Female Urban	0.78	0.66	0.92	0.92	0.86	0.99
Other Non-Poor Male Urban	1			1		

Non-overlapping confidence interval

Marginally overlapping confidence interval

Table D: Rural v/s Urban Differences in All Three Failures and CIAF in Intersectional Subgroup Comparison

	All three failures			CIAF		
		95% CI			95% CI	
Intersecting Sub-groups	OR	LCI	UCI	OR	LCI	UCI
ST Poor Female Rural	2.07	1.81	2.38	2.37	2.21	2.54
ST Poor Female Urban	1.96	0.98	3.93	1.91	1.4	2.61
ST Poor Male Rural	2.85	2.51	3.24	2.68	2.5	2.88
ST Poor Male Urban	1.94	1.2	3.15	3	2.18	4.12
ST Non-Poor Female Rural	1.43	1.08	1.89	1.34	1.18	1.52
ST Non-Poor Female Urban	1.2	0.75	1.93	1.17	0.92	1.5
ST Non-Poor Male Rural	1.95	1.52	2.52	1.52	1.35	1.72

ST Non-Poor Male Urban	1.33	0.9	1.98	1.29	1.02	1.62
SC Poor Female Rural	1.75	1.53	2	2.29	2.14	2.46
SC Poor Female Urban	2.06	1.5	2.83	2.34	1.93	2.84
SC Poor Male Rural	2.24	1.97	2.54	2.34	2.19	2.51
SC Poor Male Urban	1.93	1.44	2.59	2.48	2.03	3.03
SC Non-Poor Female Rural	0.77	0.63	0.93	1.18	1.09	1.29
SC Non-poor Female Urban	0.87	0.65	1.16	1.18	1.04	1.35
SC Non-Poor Male Rural	1.42	1.2	1.68	1.26	1.16	1.37
SC Non-Poor Male Urban	1.23	0.99	1.52	1.25	1.1	1.42
Other Poor Female Rural	1.53	1.35	1.72	2.12	2	2.24
Other Poor Female Urban	1.77	1.34	2.35	1.92	1.63	2.25
Other Poor Male Rural	2	1.78	2.24	2.07	1.95	2.19
Other Poor Male Urban	2.26	1.79	2.85	2.14	1.85	2.47
Other Non-Poor Female Rural	0.82	0.71	0.94	1.03	0.97	1.09
Other Non-Poor Female Urban	0.78	0.66	0.92	0.92	0.86	0.99
Other Non-Poor Male Rural	1.05	0.92	1.19	1.07	1.01	1.13
Other Non-Poor Male Urban	1			1		

Figure A: Univariate LISA maps of India showing clustering of undernutrition hotspot and cold spot by two dimensional and three-dimensional anthropometric failures

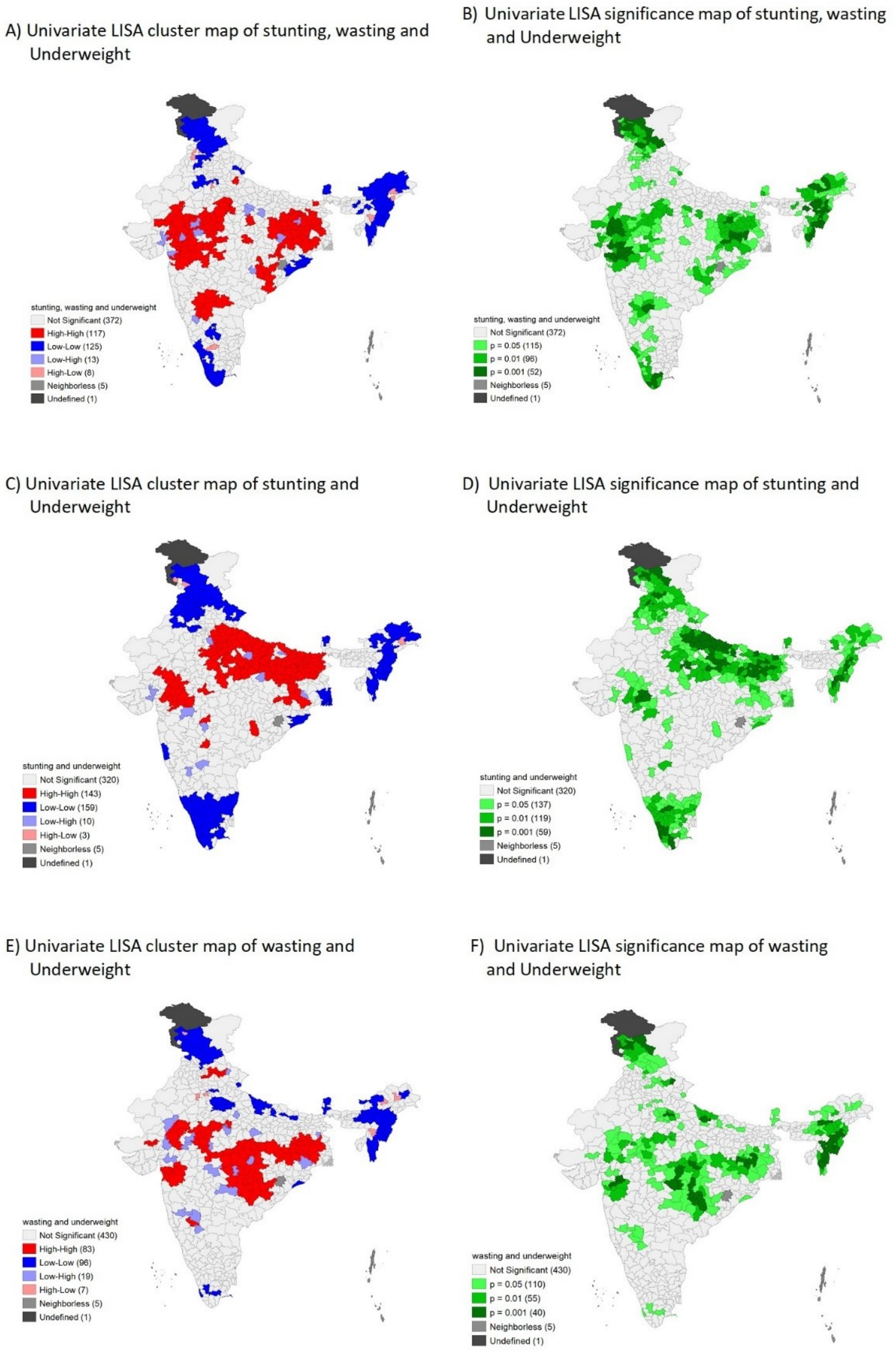

Supplement: Supplementary file 2 — Additional file 2. [file 12939_2021_1499_MOESM2_ESM.pdf]
